# Cost-effectiveness analysis of ceftazidime avibactam versus colistin in carbapenem-resistant enterobacteriaceae in Iran

**DOI:** 10.1186/s12962-023-00454-8

**Published:** 2023-07-24

**Authors:** Zahra Goudarzi, Fattaneh Danayi, Khosro Keshavarz, Ahmad Gholami

**Affiliations:** 1 Health Human Resources Research Center, Department of Health Economics, School of Health Management and Information Sciences, Shiraz University of Medical Sciences, Shiraz, Iran; 2 Faculty of Pharmacy, Shiraz University of Medical Sciences, Shiraz, Iran; 3 Emergency Medicine Research Center, Shiraz University of Medical Sciences, Shiraz, Iran; 4 Pharmaceutical Sciences Research Center, Shiraz University of Medical Sciences, Shiraz, Iran; 5 Biotechnology Research Center, Shiraz University of Medical Sciences, Shiraz, Iran; 6 Department of Pharmaceutical Biotechnology, School of Pharmacy, Shiraz University of Medical Sciences, Shiraz, Iran

**Keywords:** Cost, Effectiveness, Ceftazidime avibactam, Colistin, CRE

## Abstract

**Introduction:**

Ceftazidime avibactam (CA) is an effective treatment against carbapenem-resistant Enterobacteriaceae (CRE), but its cost-effectiveness is unclear. This study was performed to evaluate the cost-effectiveness of CA against the best available treatment colistin (Col) for patients with CRE-related infections in Iran.

**Methodology:**

A model of a decision tree was designed to evaluate the cost-effectiveness of CA in CRE patients over a period of 5 years. The Iran health system was the perspective of the study, and the discount rates of 5.8% and 3% were considered for the data of cost and utility, respectively. The clinical inputs were obtained from a prospective observational study. We established the costs of medical services and medical tariffs of Iran’s health system, and obtained the rate of medical service resources used by patients from specialists. The results of this model included the quality-adjusted life years (QALYs), increasing costs, and incremental cost-utility ratio (ICUR). We also performed the deterministic and probabilistic sensitivity analyses.

**Results:**

CA reduced the burden of related to treatment failure and the need for treatment of nephrotoxicity and chronic failure, whereas, the costs related to drug procurement and long-term care (due to longer survival) increased. Treatment with CA versus Col resulted in a 53% increase in QALYs and $425 in costs, leading to an ICUR equal to 798 $/QALYs. Sensitivity analyses proved the model’s strength and indicated that the cost-effectiveness of CA can reach 88% when paying 1111 $/QALY. Budget impact analysis estimated CA regimen will increase the health system costs by $1,270,462 in 5 years.

**Conclusion:**

In Iranian settings, CA can significantly increase the quality of life and patients’ survival; therefore, in comparison to the Col drug regimen, CA is a cost-effective strategy.

## Introduction

The global spread of carbapenem-resistant Enterobacteriaceae (CRE) is an important threat to vulnerable patients throughout the world [1, 2]. Resistance to carbapenem in Gram-negative microorganisms is of special clinical concern because carbapenems are the most effective drugs against multi-drug resistant (MDR) Gram-negative pathogens [3]. In 2017, the World Health Organization (WHO) provided a list of pathogens with global priority where CRE was considered a global priority for research and development [4].

CRE-induced bacteremia is associated with weak outcomes, such as increased length of stay and mortality. Recently, the mortality rate due to CRE bacteremia has increased by 65%, which is higher than the rate of non-CRE bacteremia (17.2%). Despite this burden, a limited number of options are available for the treatment of CRE bacteremia [5].

Studies have shown that the emergence of MDR pathogens that originate from various resources, such as humans, poultry, cow, and fish, increases the need for routine use of antibiotic sensitivity tests to identify selective antibiotics and screen the newly emerging pathogens [6].

According to the reports of laboratory activity and clinical effectiveness, colistin-based antibacterial regimens have been used as a primary treatment for CRE bacteremia. High-dose regimens of Col have been shown to increase survival and improve the treatment of CRE. However, high rates of nephrotoxicity and the complexity of sensitivity tests and dosing are among the significant disadvantages of treatment with Col, turning it into a less attractive option for the treatment of bacteremia [7].

Recently, the Food and Drug Administration (FDA) approved the use of ceftazidime-avibactam [8]. Avibactam is a non-beta-lactam inhibitor of beta-lactamase which is active against serine carbapenemase Ambler Classes A and D, such as the *Klebsiella pneumoniae* carbapenemase (KPC) and OXA-48-like carbapenemases. In contrast, avibactam does not inhibit the metallo*-β-*lactamase enzymes. Uncontrolled case series have shown variable outcomes in patients with CA-treated patients with CRE infections [9, 10].

A cohort study performed on a sufficient number of patients showed a higher treatment rate in patients receiving CA than those receiving Col (71% versus 52%). This study also showed that the mortality rate was 9% lower in the CA group [11]. On the other hand, a prospective observational study indicated that the use of CA, in comparison to Col, decreased the 30-day mortality rate. In a meta-analysis performed on the effectiveness of antimicrobial regimens, the CA regimen had more effective primary and secondary outcomes than Col. Moreover, the cost of CA drug versus Col is important [8]. As a result, the conclusion about the clinical cost-effectiveness of CA compared to Col in the treatment of CRE bacteremia is controversial. To the best of our knowledge, no study has evaluated the clinical outcomes and costs of CA versus Col in the treatment of CRE bacteremia in Iran. Therefore, this study aimed to compare the safety and cost-effectiveness of CA and Col in the treatment of CRE-induced infections.

## Methodology

### Model structure

**Fig. 1:**
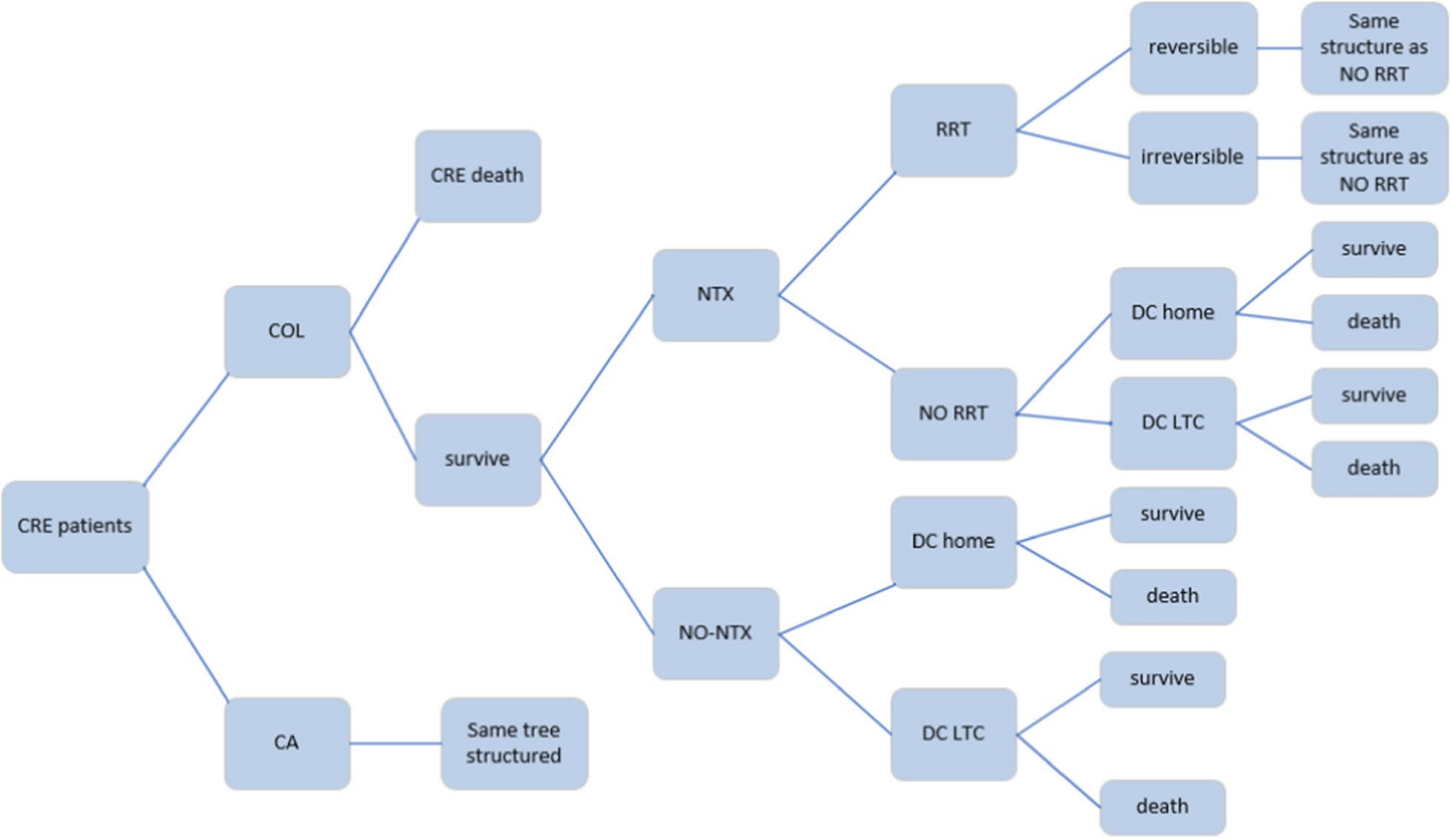
Decision tree structure for treatment of carbapenem-resistant Enterobacteriaceae (CRE). NTX, nephrotoxicity; LTC, long-term care; DC, discharge; RRT, renal replacement therapy

The cost-effectiveness analysis (CEA) model structure was designed based on a decision tree to simulate the scenarios of CA and Col in the treatment of CRE infections. A decision tree model is an appropriate tool for modeling difficulties in decision-making in acute care and midterm diseases such as infections. Figure 1 presents the model structure. A hypothetical cohort with 10,000 patients with CRE infection was considered for both groups in the model. The model was designed based on the results of effectiveness found by van Duin et al. in 5 years. The discount rates of 5.8% and 3% were considered for the data on cost and utility, respectively. The total costs and the quality-adjusted life years (QALYs) were calculated based on the occurrence of the events. Then the incremental cost-effectiveness results were calculated in 5 years to show the difference arising from the total costs and QALY of both strategies. This study was performed from the perspective of the health system.

### Study population

The target population included all patients over 18 years old with CRE-related infections. These infections were similar to that of van Duin’s study [12], in which 97% of patients suffered from *K. pneumonia*. In the CA group, the patients received 2.5 mg every 8 h, and 9 MIU (million international units) of Col was injected into the patients in the Col group once a day. The mean age of patients was 61 years, and 61% of them were male. Each patient was treated for the last time for CRE infection. The Pitt bacteremia score (PBS) was calculated based on the indicator culture day, and patients with a score of ≥ 4 were considered severely ill. A serum creatinine level of ≥ 2 mg/dL or the use of alternative medicine was defined as kidney failure.

The treatment effectiveness of CA and Col, as the comparison arm in the CRE patients, was compared in the clinical trial of van Duin and the meta-analysis of Yan Chen; the obtained data were confirmed by two specialists in Iran. In this study, these drugs were administered individually to patients. The treatment duration, mortality rate, imminent adverse effects of treatment, and possible nephrotoxicity were considered for evaluating the CA treatment’s clinical effectiveness. The possibility of renal complications in patients with nephrotoxicity was extracted from the mentioned clinical trial and confirmed by specialists.

### Utility

The data on quality of life (QoL) were extracted from reliable literature. The values of health utility considered in the model related to the following health conditions: hospitalization without nephrotoxicity, hospitalization with nephrotoxicity, chronic RRT, discharge to home, and discharge with long-term care (LTC). The utility values were obtained from previously published economic evaluations of CRE patients [7]. The length of stay of patients in the two treatment groups was obtained according to the opinions of specialists, and the duration of nephrotoxicity (28 days) and acute RRT (90 days) were estimated based on valid studies [13] (Table 1).

**Table 1:** Model inputs, ranges used in one-way sensitivity analyses, and distributions applied in probabilistic sensitivity analysis

Parameter	Value	Value lower 95% CI	Value upper 95% CI	Distribution	Source or justification
Mean age of patients	61	NA	NA	Not varied	[12]
Model time horizon	5 years	NA	NA	Not varied	[7]
Efficacy and complication for CA^a^					
Cure	0.129	0.1	0.155	Beta	[12]
Mortality at day 28	0.09	0.076	0.11	Beta	[12]
Nephrotoxicity	0.05	0.04	0.06	Beta	[12]
RRT	0.04	0.03	0.05	Beta	[12]
Discharge to home	0.559	0.44	0.67	Beta	[12]
Efficacy and complication for COL^b^					
Cure	0.07	0.056	0.084	Beta	[12]
Mortality at day 28	0.25	0.2	0.3	Beta	[12]
Nephrotoxicity	0.13	0.1	0.156	Beta	[12]
RRT	0.11	0.08	0.132	Beta	[12]
Discharge to home	0.23	0.156	0.276	Beta	[12]
Total cost					
CA based therapy	649	519	778	Gamma	Tariff^e^ of Iran's HS^f^
Col based therapy	445	356	534	Gamma	Tariff of Iran's HS
Treatment failure	808	646	969	Gamma	Tariff of Iran's HS
NTX^c^ with RRT^d^	9764	7811	11718	Gamma	Tariff of Iran's HS
NTX without RRT	901	720	1081	Gamma	Tariff of Iran's HS
Long term care	598	478	717	Gamma	Tariff of Iran's HS
Utility					
Cure	0.84	0.756	0.924	Beta	[7]
No cure	0.73	0.657	0.803	Beta	[7]
Nephrotoxicity	0.66	0.594	0.726	Beta	[7]
Long-term care	0.64	0.576	0.704	Beta	[7]
Chronic dialysis	0.59	0.531	0.649	Beta	[7]
Discount rate					
Cost	0.058	NA	NA	Not varied	[22]
QALY	0.03	NA	NA	Not varied	[22]

a: ceftazidime-avibactam, b: colistin, c: nephrotoxicity, d: renal replacement therapy, e: https://ta.muq.ac.ir, f:health system

### Cost input

The costs considered in the CEA model were medicinal treatment, management of infections during hospitalization, chronic RRT, LTC of costs related to the drug administration, and nephrotoxicity. The treatment costs at the beginning of the model, all patients received CA or Col based on the treatment group. The prices of Col and of CA were obtained from the least price reported by the Iranian FDA and the price proposed by the Jaber Ebne Hayyan Company to enter the national official list, respectively. All other data, such as dose, mean treatment duration, and distribution of patients in various treatment options, which were required for the calculation of the treatment costs were obtained from the observational study of van Duin [12]. The hospital costs were estimated from national tariffs and included all costs imposed during hospitalization. The cost of disease complications of this model assumes that after the failure of the treatment, patients receive a second course of antibiotics. The medical services provided to patients with chronic RRT and LTC (including hospitalization, dialysis, kidney transplantation, diagnostic methods, and drugs) were extracted from an interview with infectious disease specialists and urologists, and the annual costs of these services were calculated based on national tariffs (Table 1).

### Sensitivity analysis

A deterministic sensitivity analysis *(*DSA) and a probabilistic sensitivity analysis (PSA) were performed to evaluate uncertainty in the model’s parameters. DSA was carried out to assess the effect of any changes in the parameter on the results of estimated ICER, and PSA to assess the affectivity of all parameters in a Mont Carlo simulation with 10,000 people. The results of PSA were used to develop a cost-effectiveness acceptability curve (CEAC) to evaluate the possibility of acceptability of each treatment strategy. The gamma distribution was considered for continuous and positive variables (costs and length of stay) and the beta distribution for variables that considered values between 0 and 1 (i.e., possibility, utility) [14]. CEAC can indicate the cost-effectiveness of a treatment based on the values and uncertainty of the parameters used in the model and for different values of acceptable WTP. Finally, an alternative scenario analysis was also performed based on the time horizon.

### Budget impact analysis

We developed a decision tree-based model of budget impact to estimate the direct medical costs for patients with CRE in case of access to DRD treatment, according to the viewpoint of Iran’s health system. The present treatment scenario (without CA) was compared with the future scenario (with CA) in 5 years.

According to the physicians’ opinions and literature, the annual hospitalization rate in Iran is about 0.006 [15]. In addition, a comprehensive meta-analysis in Iran estimated the nosocomial infection rate as 4.5%. Moreover, Nasiri et al. showed that 29% of these cases suffer from CRE [16]. According to this prevalence rate, we estimated the number of patients with CRE in Iran as 6627 people in 2021. In this model and based on physicians’ opinions, we assumed that about 40% of these patients are eligible to receive both CA and Col regimens; about 2651 people in Iran. In this model, the sales volume pattern of Col and other alternative drugs for the treatment of hospital-acquired pneumonia was estimated considering their trend in previous years. To this end, we evaluated the data on the sales volume of each drug mentioned in the annual pharmaceutical statistics (2017–2022). Then we inserted the sales volume data in Excel and predicted the pattern of market share growth for each drug until 2016. The results showed that the market share of Col will increase from 17% to 2021 to 27% in 2027. In addition, we assumed that CA will allocate 1% of the market share in the first year and will increase to 5% by 2027. Drugs, diagnostic services, long-term care, nephrotoxicity-related care, RRT, visit, relevant AEs, and hospitalization are the main factors that can affect the budget. We did not consider the inflation rate in the health system costs in the upcoming years.

## Results

### Base-case results

Our base-case analysis showed that during a 5-year time horizon, the CA group earned 1.71 QALY by spending $885. However, the Col group earned 1.18 QALY by spending $460 during this period; meaning that CA-receiving patients earned 0.53 more QALY. ICER obtained from Markov analysis was 798 $/QALY which is lower than the $1111 threshold in Iran (The threshold in Iran is 400 million Rial, which was adjusted on November 1, 2022 at the rate of one dollar, equivalent to 360,000 Rial). Furthermore, NMB for the CA treatment strategy was higher than the Col treatment, indicating that considering the acquired effectiveness, the value of spent money on the CA treatment was higher and will have a higher financial value. The results of ACER showed that higher financial resources are required for earning 1 QALY in the CA group (Table 2).

**Table 2:** Results of base case analysis

Parameters	Col group	CA group	Increment
Cost	460	885	425
QALY	1.18	1.71	0.53
ICER	-	-	798
ACER	390	517	127
NMB	849	1015	166

### Sensitivity analysis

**Fig. 2:**
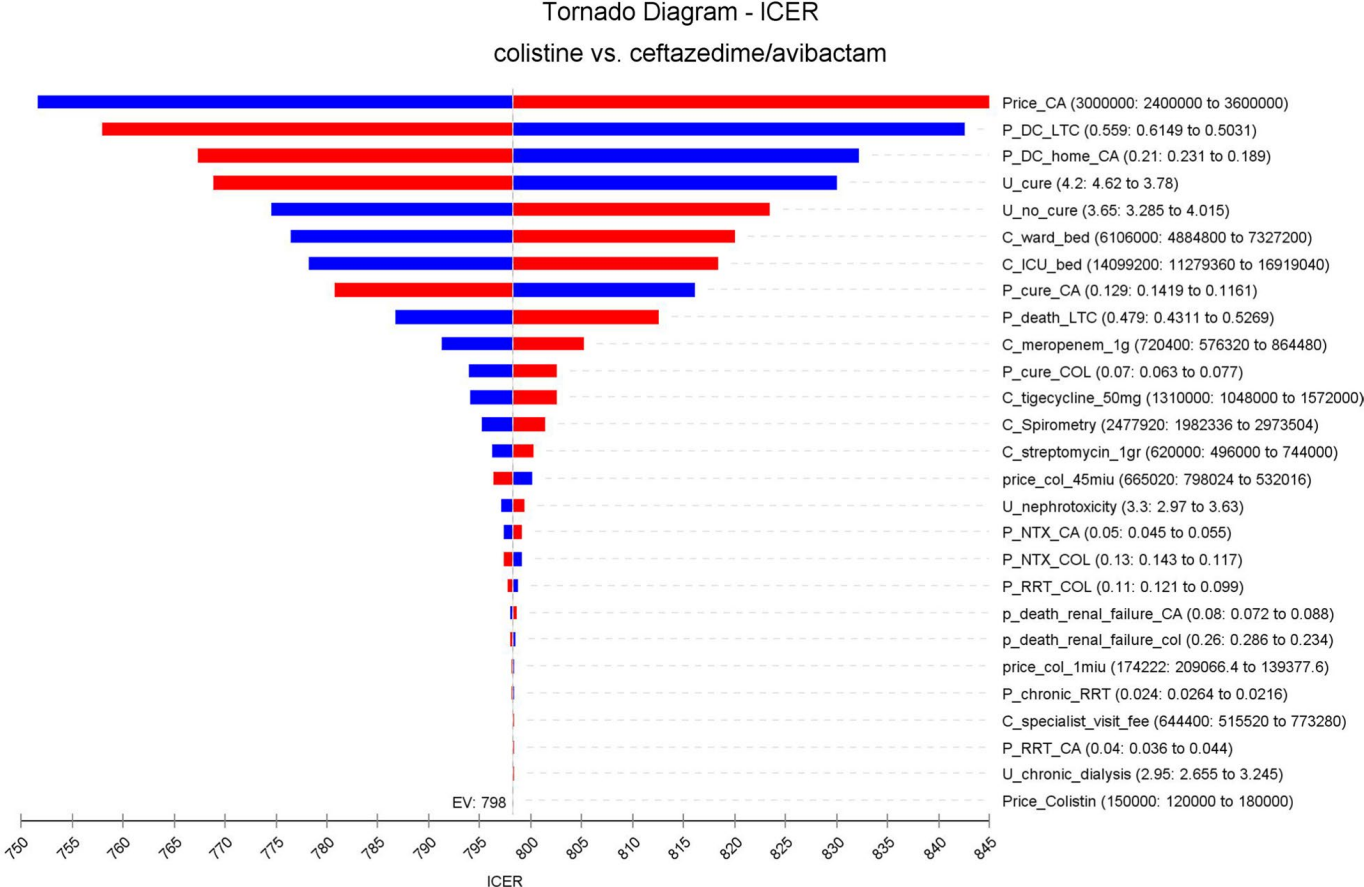
Tornado analysis depicting results of one-way sensitivity analysis

**Fig. 3:**
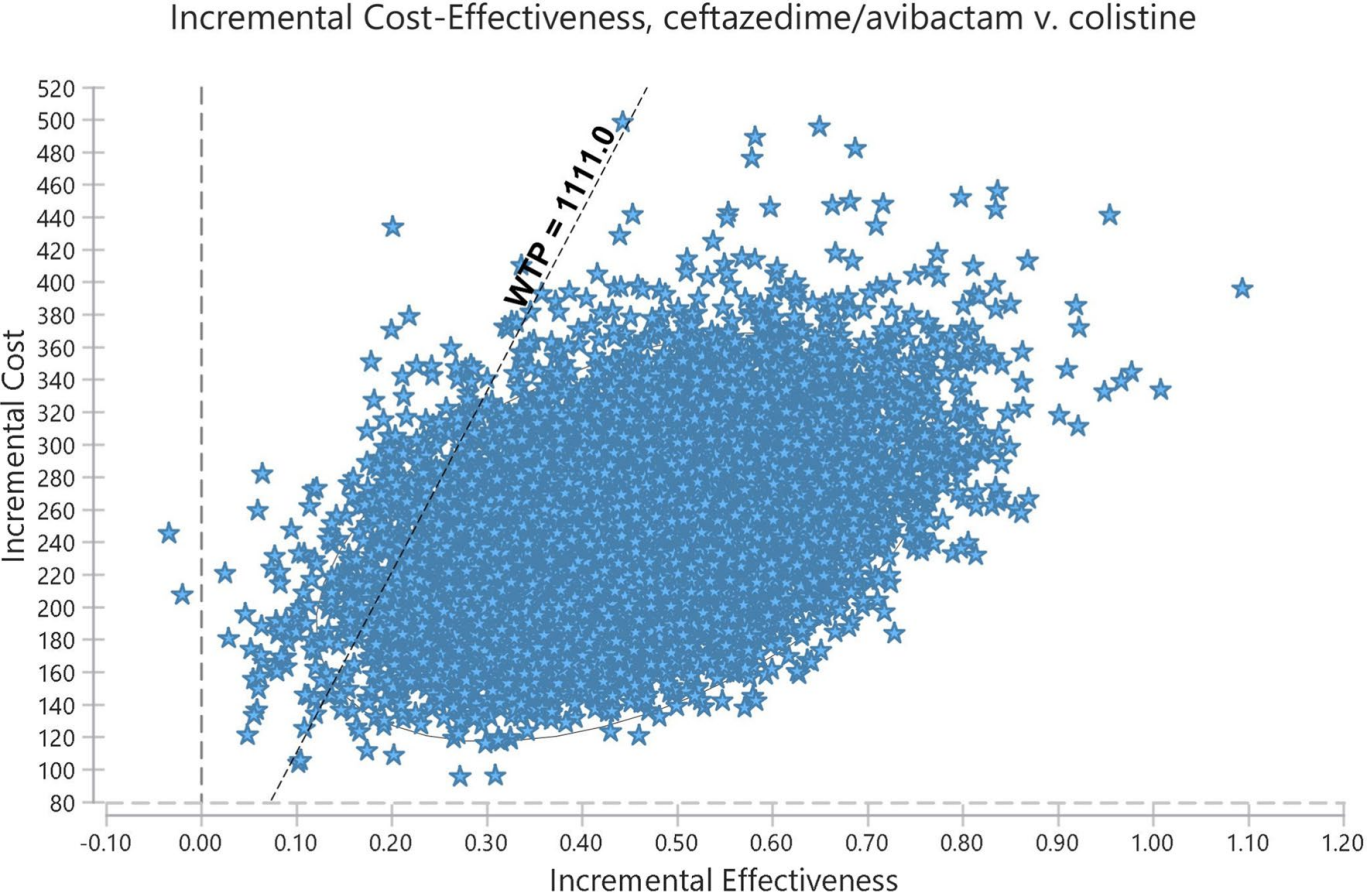
probabilistic sensitivity analysis

**Fig. 4:**
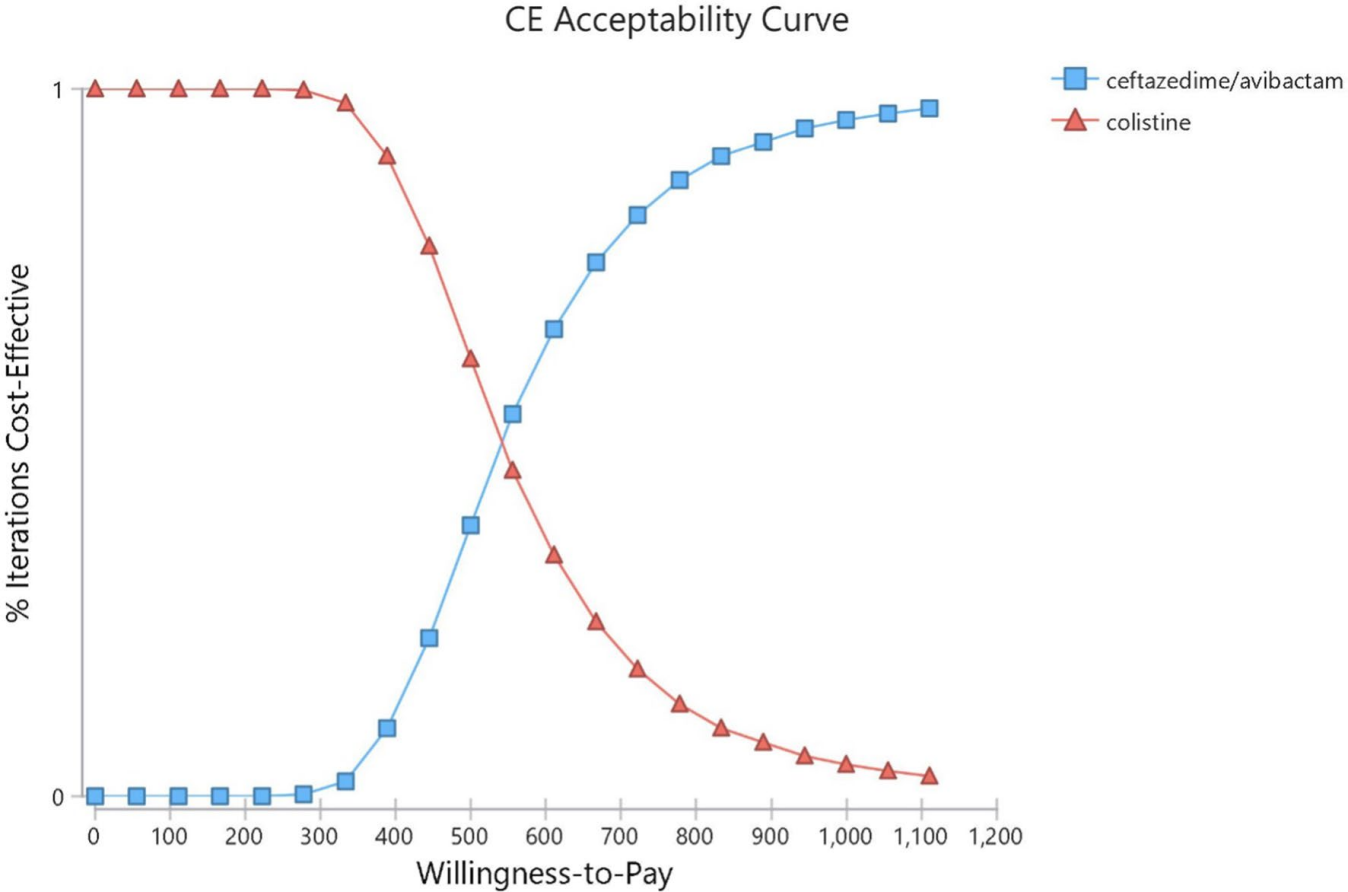
cost-effectiveness acceptability curve

The deterministic sensitivity analysis (DSA) showed that the CA price is the most important factor that can affect the scenario analysis (Fig. 2). Other variables, such as the possibility of patients’ discharge or the treatment utility can result in many changes in ICER, but these changes had no significant effect on the study results because ICER will remain under the threshold. The results of probabilistic sensitivity analysis (PSA) are shown in Fig. 3. According to this diagram, CA will produce more QALYs than Col in the given distributions because the SD of CA regimen cost is higher than Col. In total, in the given distributions, the CA strategy was superior to the Col strategy in 88% of cases. The cost-effective acceptance curve shows that by increasing of WTP rate, the acceptance rate of CA treatment increased (Fig. 4).

### Budget impact analysis

Budget impact analysis estimated that after access to the CA treatment, the increase in the costs in the health system is expected to be 29%, 34%, 38%, 42%, and 46% during the five years, respectively. In other words, adding the CA regimen to the therapeutic basket of patients with CRE will increase the health system costs by $1,270,462 in 5 years. Table 3 presents the budget impact on the public health financial burden from 2023 to 2027.

**Table 3:** Results of budget impact analysis

Year	2023	2024	2025	2026	2027
Iran population	86,369,815	87,406,253	88,455,128	89,516,589	90,928,011
number of patients of CRE	6788	6869	6952	7035	7120
total number of CRE patients who deserve to receive CA or COL	5430	5495	5561	5628	5696
COL market share	0.24	0.25	0.26	0.26	0.27
CA market share	0.01	0.02	0.03	0.04	0.05
Scenario 1(without CA)	598,622	636,466	665,679	686,422	708,170
Scenario 2(with CA)	777,117	853,953	920,706	977,588	1,036,456
Financial impact	178,495	217,487	255,027	291,166	328,287
Financial impact%	29%	34%	38%	42%	46%

## Discussion

Considering the obtained ICER 798 $/QALY that is much lower than the accepted threshold of 1111 $/QALY, treatment of CRE-related infection with CA is a cost-effective option from the viewpoint of the health system. In general, the cost-effectiveness model in this study estimated that the incremental costs relating to CA versus Col treatment in a representative patient with CRE-KPC infection are $798 in a 5-year horizon. This increment is mainly due to the cost of pharmacotherapy and LTC related to higher survival. Especially, a higher cost of LTC is due to higher survival of CA-treated patients. On the hand, it is expected that costs associated with non-treatment and RRT decrease due to an increase in the efficiency and safety of CA in comparison with Col. In terms of effectiveness, the model estimated an increase in QALYs by 0.53. The increase in QALYs is mainly associated with an increase in survival after discharge, a decrease in the length of stay along with nephrotoxicity, and a decrease in the need for RRT. Although a limited number of economic studies have been performed in this regard, the results of this study are completely consistent with that of the study of Varón-Vega et al. [17]. In addition, the study of Kongnakorn, which compared the cost-effectiveness of CA versus imipenem in patients with urinary tract infection (UTI), showed that CA is a cost-effective regimen [18]. Shildz et al. obtained better results for CA than Col in terms of clinical success (85% versus 40%) and 30-day mortality (8% versus 30%) [19]. Tumbarlo et al. showed that CA had a lower mortality rate in comparison with other life-saving regimens for CRE infections (36.5% versus 55.8%) [20].

This study was performed from the viewpoint of the Iran health system; therefore, its results can be used for prioritizing or allocating resources. These results can be reinforced by improving strategies such as antibiotic surveillance to reduce bacterial resistance and optimize the use of antibiotics. In Iran, the consumption of antibiotics is significantly high. As a result, similar studies and relevant guidelines can help more effective use of antibiotics, such as vancomycin, third-generation cephalosporins, aminoglycosides, and carbapenems, and reduce the rate of healthcare-related infections.

This model showed that, in comparison with Col, CA can provide a better opportunity for clinical success by decreasing the number of people that require additional antibiotics, reducing the mortality rate, and increasing QALY. Despite its clinical advantages, CA does not save money for patients. This difference is mainly due to the difference in the prices of these two drugs so that the higher price of CA versus Col and longer survival of CA-treated patients do not lead to further use of resources in these patients because the absolute difference in the mortality rate was 23%. This study showed that considering the direct medical costs, patients who survived the infection stayed longer in the hospital and had a higher cost.

There is a debate regarding the difference in effectiveness and safety of Col and CA, which mainly pertains to the pharmacokinetics of obtaining Col from colistin methane sulfonate [21]. Based on this uncertainty, a systematic study that compared both treatments showed a significant difference in the 30-day mortality rate between these two strategies. In addition, Col was associated with worse secondary outcomes such as nephrotoxicity and relapse of the disease [8].

To the best of our knowledge, this is the first study into the cost-effectiveness of CA for the treatment of CRE-KPC-related infections, although it faced some limitations. First, only the direct costs that were provided by the health system were considered in this study, and the indirect costs were omitted, for example, the reduced efficiency of patients and their caregivers that is expected to reduce by a decrease in the disease complications. Second, due to the lack of use of CA in Iran, we had to estimate the utility and effectiveness values based on valid references. Third, nephrotoxicity is a critically adverse effect of some drugs which was considered. Fourth, the lack of randomized trials in this regard that reflect the effectiveness of comparative treatments was another limitation of this study. With regard to these weaknesses, the data of this study are based on an observational study with some limitations in the design and nature. One of the reasons for the lack of such trials is inaccessibility to people with multi-drug-resistant infections. On the other side, this observational study had some strengths, for example, it had an acceptable sample size (n = 137), it was prospective and was performed in several hospitals, and it followed patients for a long time. Due to uncertainty in some data, the clinical inputs of the model were evaluated through probabilistic sensitivity analysis which showed that CA was more cost-effective than Col in 88% of the cases.

## Conclusion

The cost-effectiveness model shows that, in comparison to colistin, ceftazidime-avibactam leads to an increase in the quality of life and a decrease in death cases, kidney failure, and treatment failure which finally results in a cost-effective treatment for carbapenem-resistant *K. pneumonia*.

## Data Availability

All data are available on reasonable request.

## References

[1] Gomez-Simmonds A (2016). Combination regimens for treatment of carbapenem-resistant Klebsiella pneumoniae bloodstream infections. Antimicrob Agents Chemother.

[2] Falcone M (2016). Predictors of outcome in ICU patients with septic shock caused by Klebsiella pneumoniae carbapenemase–producing K. pneumoniae. Clin Microbiol Infect.

[3] Strich JR (2021). Pharmacoepidemiology of ceftazidime-avibactam use: a retrospective cohort analysis of 210 US hospitals. Clin Infect Dis.

[4] Tacconelli E (2018). Discovery, research, and development of new antibiotics: the WHO priority list of antibiotic-resistant bacteria and tuberculosis. Lancet Infect Dis.

[5] Hakeam HA (2021). Effectiveness of ceftazidime–avibactam versus colistin in treating carbapenem-resistant Enterobacteriaceae bacteremia. Int J Infect Dis.

[6] Snyman Y. 2022-09-10 Colistin resistance in Gram-negative pathogens in the Western Cape, South Africa. 2021.

[7] Simon MS (2019). Cost-effectiveness of ceftazidime-avibactam for treatment of carbapenem-resistant *Enterobacteriaceae* bacteremia and pneumonia. Antimicrob Agents Chemother.

[8] Chen Y (2022). Efficacy and safety of ceftazidime-avibactam for the treatment of carbapenem-resistant *Enterobacterales* bloodstream infection: a systematic review and meta-analysis. Microbiol Spectr.

[9] Voulgaris GL, Voulgari ML, Falagas ME (2019). Developments on antibiotics for multidrug resistant bacterial gram-negative infections. Expert Rev anti-infective therapy.

[10] Farina D (2014). The inhibition of extended spectrum β-lactamases: hits and leads. Curr Med Chem.

[11] Almangour TA (2022). Ceftazidime-avibactam versus colistin for the treatment of infections due to carbapenem-resistant Enterobacterales: a multicenter cohort study. Infect Drug Resist.

[12] Van Duin D (2018). Colistin versus ceftazidime-avibactam in the treatment of infections due to carbapenem-resistant Enterobacteriaceae. Clin Infect Dis.

[13] Prescott GJ (2007). A prospective national study of acute renal failure treated with RRT: incidence, aetiology and outcomes. Nephrol Dialysis Transplant.

[14] Rahman G et al. On-Gamma and-Beta Distributions and Moment Generating Functions. Journal of Probability and Statistics, 2014. 2014.

[15] Rouhani S (2021). The impacts of family physician plan and health transformation plan on hospitalization rates in Iran: an interrupted time series. BMC Health Serv Res.

[16] Nasiri MJ (2020). Prevalence and mechanisms of carbapenem resistance in *Klebsiella* pneumoniae and *Escherichia* coli: a systematic review and meta-analysis of cross-sectional studies from Iran. Microb Drug Resist.

[17] Varón-Vega F (2022). Cost-utility analysis of ceftazidime-avibactam versus colistin-meropenem in the treatment of infections due to Carbapenem-resistant Klebsiella pneumoniae in Colombia. Expert Rev PharmacoEcon Outcomes Res.

[18] Kongnakorn T (2019). Cost-effectiveness analysis of ceftazidime/avibactam compared to imipenem as empirical treatment for complicated urinary tract infections. Int J Antimicrob Agents.

[19] Shields RK (2017). Emergence of ceftazidime-avibactam resistance due to plasmid-borne bla KPC-3 mutations during treatment of carbapenem-resistant Klebsiella pneumoniae infections. Antimicrob Agents Chemother.

[20] Tumbarello M (2019). Efficacy of ceftazidime-avibactam salvage therapy in patients with infections caused by Klebsiella pneumoniae carbapenemase–producing K. pneumoniae. Clin Infect Dis.

[21] Garonzik S (2011). Population pharmacokinetics of colistin methanesulfonate and formed colistin in critically ill patients from a multicenter study provide dosing suggestions for various categories of patients. Antimicrob Agents Chemother.

[22] Mozayani AH, Sahabi B, Asadi M (2021). Estimating social discount rate trend in Iran. Iran Economic Rev.

